# Effectiveness of an Internet-Based Self-Help Acceptance and Commitment Therapy Program on Medical Students’ Mental Well-Being: Follow-Up Randomized Controlled Trial

**DOI:** 10.2196/50664

**Published:** 2024-12-04

**Authors:** Difan Wang, Bingyan Lin, Shuangxi Zhang, Wei Xu, Xinying Liu

**Affiliations:** 1 Department of Internal Medicine, Psychological Counseling and Service Center Graduate School of Medical College of Chinese PLA General Hospital Beijing China; 2 Faculty of Psychology Beijing Normal University Beijing China; 3 Department of Primary and Long-term Care University Medical Center Groningen University of Groningen Groningen Netherlands; 4 School of Education Hebei University Baoding China; 5 Department of Otolaryngology Head and Neck Surgery The Third Medical Center of Chinese PLA General Hospital Beijing China

**Keywords:** internet-delivered self-help acceptance and commitment therapy, depression, anxiety, stress, psychological inflexibility, obsessive-compulsive symptoms, medical students, iACT 2.0 program

## Abstract

**Background:**

Psychological distress is a growing problem among medical students worldwide. This highlights the need for psychological interventions to focus on mental health and improve well-being in this population.

**Objective:**

This study developed an internet-based, self-help, acceptance and commitment therapy program (iACT 2.0), aiming to examine its effectiveness in reducing depression, anxiety, stress, psychological inflexibility (PI), and obsessive-compulsive symptoms (OCSs) among medical students.

**Methods:**

A total of 520 Chinese postgraduate medical students were randomly assigned to either an iACT 2.0 intervention group (n=260; six online lessons, once every 5 days) or a control condition (n=260; without intervention). Participants completed questionnaires including the 21-item Depression Anxiety Stress Scale, the revised Obsessive-Compulsive Inventory, and the Multidimensional Psychological Flexibility Inventory at the preintervention (T1), postintervention (T2), and 1-month follow-up time points (T3). No therapist support was provided during the 1-month iACT 2.0 intervention period. Data were collected via an online platform and analyzed using repeated-measures ANOVA.

**Results:**

Participants in the intervention group demonstrated a significant decrease in depression, anxiety, stress, PI, and OCSs compared to the control group after the intervention (*F*=22.9-672.04, all *P*<.001). Specifically, the intervention group showed significant reductions in all measured outcomes from the preintervention to postintervention time point and at the 1-month follow-up (all *P*<.001). In contrast, no significant changes were observed in the control group over the same period (all *P*>.05). The groups did not differ significantly at baseline (all *P*>.05). Significant differences were noted at both the postintervention and follow-up time points (all *P*<.001).

**Conclusions:**

This study demonstrated that the newly developed iACT 2.0 was effective in reducing depression, anxiety, stress, PI, and OCSs. Notably, the positive effects of the intervention persisted at the 1-month follow-up. This program can offer a useful addition to existing mental illness treatment and lead to improvements in clinical and psychotherapy planning while simultaneously reducing the burden on traditional counseling and services.

**Trial Registration:**

Chinese Clinical Trial Registry ChiCTR2300070725; https://tinyurl.com/2h75wx8n

## Introduction

### Background

Psychological distress (depression, anxiety, and stress) is highly prevalent among medical students, raising significant concerns [1-3]. Research indicates that medical students experience significantly higher levels of anxiety, depression, and stress compared to their age-matched peers in the general population [4]. For example, a meta-analysis revealed that approximately 1 in 3 medical students worldwide experiences anxiety, a rate significantly exceeding that of the general population [5]. Moreover, medical students exhibit a notable prevalence rate of 27.2% for depression, compared to 9.3% in the general populace [6]. The demanding journey through medical school frequently triggers substantial stress and anxiety [7-10]. Anxiety involves anticipating future threats, often accompanied by muscle tension and heightened vigilance, while depression is characterized by feelings of sadness or emptiness, impairing effective functioning [11]. Stress, defined as feeling unable to meet requirements or expectations, manifests in physical, psychological, or social complaints [12,13].

Previous studies highlight obsessive-compulsive disorder (OCD) as a significant mental health concern among Chinese medical students [14]. The prevalence of symptoms suggestive of OCD among medical students surpasses that of the general population and is correlated with depressive symptoms [15]. Additionally, the COVID-19 pandemic has exacerbated OCD prevalence among medical students [16,17]. Additionally, enforced prevention measures such as repeated disinfection and strict hand hygiene heighten their sense of compulsion. OCD is characterized by the presence of obsessions and compulsions. Obsessions refer to recurrent and persistent thoughts, urges, or images that are intrusive and undesired, whereas compulsions are repetitive behaviors or mental actions that an individual feels driven to perform in response to an obsession or according to rules that must be applied rigidly [11]. Therefore, based on existing literature, depression, anxiety, stress, and OCD are prominent emotional distresses among medical students, warranting further attention.

Psychological distress presents potential risks for both patients and medical students, raising concerns about mental health challenges within this group [2]. Poor mental health can lead to unprofessional behavior, including difficulties in decision-making and increased errors [18,19], reduced attention and concentration [19], and loss of empathy [20], ultimately affecting education, employment, and social relationships and causing poorer quality of life, lower self-esteem, and a sense of hopelessness [21]. Moreover, OCD can seriously interfere with an individual’s social and occupational functioning, potentially affecting academic performance [22,23], social interactions, and cognitive abilities [24], thereby impeding normal life and work.

Despite the high prevalence and risk of psychological distress among medical students, many medical students with mental health problems do not receive treatment. Research indicates that only 22% of medical students who are depressed actually access mental health services [4]. Common barriers that prevent students from using these services include time constraints, limited accessibility, concerns about confidentiality, stigma, fear of unwanted interventions, and cost [4,25]. Consequently, it is crucial to use all available resources to develop sound innovations to tackle these challenges effectively.

### Potential of Acceptance and Commitment Therapy in the Treatment of Mental Illness Among Medical Students

While medical students face challenges in managing mental health problems, and receive limited and inadequate behavioral health education and training [26,27], studies indicate that they have better recognition of mental illnesses compared to students in other disciplines [28,29]. Acceptance and commitment therapy (ACT) emphasizes the willingness to experience unwanted thoughts and emotions and contact with the present moment rather than avoid emotional distress [30], and it involves mindfulness and acceptance processes and behavior change processes [31]. The primary goal of ACT is to improve psychological flexibility (PF)—the ability to pursue value—and live a meaningful life in the presence of discomfort and other unwanted inner experiences [30,32].

A growing body of evidence indicates that ACT can alleviate depressive symptoms, anxiety, stress, and emotional distress in various populations [31,33,34]. For instance, prior meta-analyses have shown that ACT yields small to medium effects in reducing depressive symptoms, anxiety, and stress among family caregivers [34]. Positive mental health outcomes have also been observed among university students [35,36], parents [37], adolescents [38], and others. Moreover, ACT has demonstrated effectiveness in addressing psychological distress while achieving high satisfaction rates [39,40]. Notably, Pang et al [41] conducted a small-scale randomized controlled trial (n=22) involving clinical medical students in Borneo to assess the efficacy of a 1-day, therapist-delivered ACT intervention in alleviating performance anxiety. The intervention group exhibited significant improvements in anxiety immediately after the intervention and at the 1-month follow-up, indicating the potential for conducting a larger randomized controlled trial to verify the efficacy of ACT among medical students.

Furthermore, ACT is one of the newest treatment strategies that has been rapidly developed to improve the treatment of patients with OCD, by teaching individuals to shift their relationship with obsessive thinking rather than resist or reduce the number of obsessions and compulsions [42-44]. Studies have consistently found that ACT can improve the PF of patients with OCD, which in turn predicts the reduction of obsessive-compulsive symptoms (OCSs) [45-47]. Furthermore, ACT has been shown to be as effective as drug therapy in improving the quality of life of patients with OCD [42].

### Advantages of Internet-Based Interventions

Internet-based psychological interventions have gained popularity and proven to be beneficial [48-50]. Notably, ACT-based, internet-delivered treatments have been developed for health anxiety [51,52] and internet-based cognitive behavioral therapy has been used for OCD treatment [53]. Online interventions offer benefits such as breaking distance and time limits [54], providing more privacy and flexibility [36], and allowing access from anywhere at any time for rural medical students [7].

Although previous systematic reviews and meta-analyses have reported the effectiveness of internet-based ACT (iACT) programs in reducing depressive symptoms, anxiety, stress, and psychological distress across diverse populations [31], few studies explore the impact of iACT interventions on medical students, particularly those in China. Given their familiarity and regular use of digital technology and the internet for accessing health-related information [55], there is a compelling rationale for developing a highly feasible and engaging intervention program that can be perceived as a means of enhancing the mental health of Chinese medical students.

### Objective and Our Study

Cultural adaptation involves the systematic modification of an evidence-based treatment to align with the client’s language, culture, and context, ensuring compatibility with their cultural values, including factors such as interdependence and spirituality [56,57]. Research consistently shows that culturally adapted treatments are more effective, acceptable, and satisfying for patients [58-60]. Studies have also demonstrated that participants find culturally adapted ACT enjoyable and acceptable [58,61,62]. Therefore, considering cultural adaptation can be highly beneficial in our ACT interventions.

Furthermore, traditional ACT typically requires multiple sessions to systematically explore various facets of PF and psychological inflexibility (PI), which can impose significant economic and time constraints. Research has shown that these constraints often serve as barriers for medical students in accessing psychological services [4,25]. Given the proven effectiveness of single-session counseling [63], it is both logical and timely to develop an intervention that has the potential to evolve into a single-session therapy in the future.

In this study, we developed an internet-based, self-help ACT program (iACT 2.0) by incorporating cultural and spiritual elements into traditional ACT. Each session follows the 4 steps of “启-承-转-合” (means starting-proceeding-transforming- integrating in Chinese) to illustrate the process of PF and PI, with each step representing its corresponding therapeutic goal within a single session. Additionally, we used the Bagua diagram from traditional Chinese Daoism to illustrate these 4 steps, symbolizing the continuous change and thought patterns, thereby enhancing the relatability and comprehensibility of the intervention. Unlike traditional ACT, we introduced all 6 facets in the first lesson and used subsequent sessions to reinforce core ACT concepts with diverse examples. This approach encourages participants to initially focus on their most inflexible facet and potentially find solutions during the first session, facilitating problem-solving and promoting flexibility through internal exploration. The core content was delivered in 6 online lessons, spaced 5 days apart, to systematically reinforce PF. To maximize engagement, participants were asked to practice daily mindfulness during the intervention. Importantly, no direct therapist interaction was included, allowing us to isolate the effects of the online program itself. Self-guided internet-based therapies have shown positive results in treating depression [64]. In conclusion, our intervention was designed to enhance accessibility and acceptance for participants.

To the best of our knowledge, this study represents the pioneering application of iACT to a cohort of Chinese postgraduate medical students. The objective of our study was to evaluate the efficacy of iACT 2.0, an internet-based, self-help ACT program, in reducing depression, anxiety, stress, OCSs, and PI in medical students. We hypothesized that iACT 2.0 would yield significant improvements in primary outcomes when compared to the waitlist controls, with these improvements sustained for a 1-month follow-up.

## Methods

### Study Design

This study was designed as a single-blinded randomized controlled trial consisting of 2 parallel groups with a blocked 1:1 allocation. During recruitment, all participants were informed about the intervention but not their group assignment, ensuring their unawareness of group allocation throughout this study. Maintaining participant blinding was crucial to prevent potential bias in questionnaire responses, particularly for those in the control group. The randomization was performed by a research assistant who was not part of this study. The researcher remained unaware of group assignments until after finalizing randomization. The trial was conducted from April 2023 to June 2023. To ensure adherence to randomized controlled trial guidelines, the trial was registered in the Chinese Clinical Trials Registry under the registration ChiCTR2300070725.

### Participants

Medical students from the Graduate School of Medical College of Chinese PLA Hospital were recruited in February 2023 via WeChat (Tencent) advertisements, stating that there is a newly developed psychotherapy program aimed at reducing psychological distress. It also stated that the intervention would be delivered via the internet and that participants would be expected to practice daily. Participants can participate in this study by searching the name of our WeChat Mini Program and then logging in to the platform to complete an online screening. Sign-up for this study was open from April 15 to 23, 2023. This study’s inclusion criteria were (1) being a postgraduate student enrolled at the Graduate School of Medical College of Chinese PLA Hospital; (2) being aged 18 years and older; and (3) having 21-item Depression Anxiety Stress Scale (DASS-21) scores indicating at least mild depression, anxiety, and stress levels (depression subscale ≥10, anxiety subscale ≥8, and stress subscale ≥15) [65,66]. The exclusion criteria were (1) individuals who were currently receiving psychotherapy, (2) organic mental disorder or schizophrenia, (3) visual or hearing impairment, (4) severe drug or alcohol use disorders, and (5) pregnant and lactating women.

A total of 563 students expressed interest in participating in this study. Before the preintervention measurement, all participants were provided with an online written consent form to review and sign. On review of eligibility criteria, 43 participants were excluded from this study, leaving a final sample of 520 participants. Participants were randomly assigned to either the iACT 2.0 intervention group (iACT group, n=260) or the waitlist control group (control group, n=260) at a 1:1 allocation ratio. The randomization process was conducted by an independent individual, unaffiliated with this study, who used computer-generated pseudorandom numbers to allocate participants, ensuring the concealment of allocation from participants.

In total, 17 participants in the iACT group and 14 participants in the control group did not complete the posttest, while 3 participants in the iACT group and 8 participants in the control condition did not complete the follow-up test, Therefore, the final number of participants in this study was 478, with a dropout rate of 8% (42/520). The flow diagram is presented in the *Results* section.

### Procedures

Participants in this study were assessed at the preintervention (T1), postintervention (T2), and 1-month follow-up time points (T3). The pre- to postintervention comparison investigated whether the iACT 2.0 had been effective in comparison to the no-intervention control condition (control). The 1-month follow-up studied the maintenance of the intervention effects. At the preintervention measurement, participants were asked to fill out the relevant online questionnaires and sociodemographic information. When the preintervention measurement was finished, participants in the intervention group were given access to the online intervention lesson, including the link, username, and password. The postintervention measurement was conducted 30 days after the start of the intervention by filling in the questionnaires. Participants were asked to complete the same questionnaires again for the 1-month follow-up measurement. Control participants were given access to the intervention lesson after the follow-up test.

### Intervention

**Figure 1:**
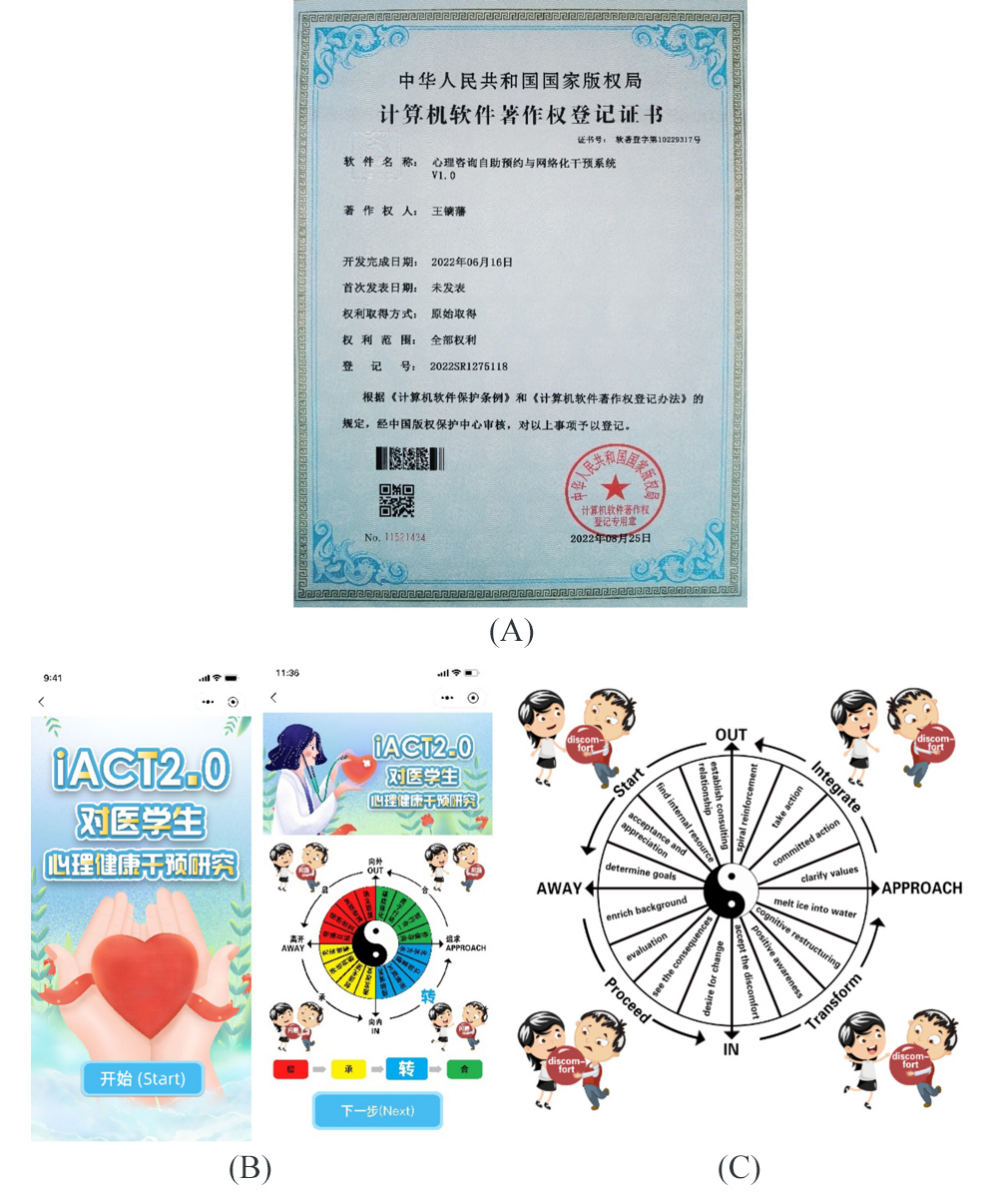
(A) The copyright registration certificate for our intervention system; (B) screenshot of the program; (C) English translation version (see Figure S1 in Multimedia Appendix 1 for the English version).

The 1-month internet intervention (Textbox 1; see Table S1 in Multimedia Appendix 1 for detailed intervention protocol) was delivered through a patented online system (Figure 1A and B), without any personal support provided. The intervention was based on the 6 facets of ACT and consisted of 6 online lessons delivered in various media formats, including text, video, audio, and illustrations. Participants in the iACT group were instructed to practice daily mindfulness for 30 days (20-30 minutes per day) to increase their awareness. The lessons were released sequentially, with access to the next lesson granted on completion of the previous lesson. The control group did not receive any intervention.

The intervention process was designed to align with the principles of ancient Chinese Daoism and used the 4 steps of “启-承-转-合” (which means starting-proceeding-transforming- integrating in Chinese; Figure 1C). This iterative process introduced the 6 facets of PF and PI in the initial lesson, followed by increasingly sophisticated examples of these facets in subsequent lessons. Participants were instructed to proactively identify areas of PI and select the appropriate facet to apply to their daily lives. This process enabled participants to enhance their self-awareness and cultivate greater PF through the application of ACT principles.

**Overview of the internet-based, self-help, acceptance and commitment therapy program (iACT 2.0) treatment content showing time and content summary.**

**Lesson 1 (day 1)**

- Start: Set objectives.
- Proceed: Review their previous coping styles.
- Transform:
  Elucidate the 6 facets of psychological flexibility (PF) and psychological inflexibility (PI) of acceptance and commitment therapy (ACT).
  Mindfulness practice: body scan in mindfulness.
- Integrate: Choose one facet of PF and use it as a coping strategy in the following 5 days.
- Homework: Practice mindfulness (body scan) daily until the next lesson.

**Lesson 2 (day 6)**

- Start: Review the coping style of the past 5 days.
- Proceed: Think about the long-term effects of previous coping manners.
- Transform:
  Elucidate the 6 facets of PF and PI of ACT with different examples.
  Mindfulness practice: body stretch.
- Integrate: Choose one facet of PF and use it as a coping strategy in the following 5 days.
- Homework: Practice mindfulness (body stretch) daily until the next lesson.

**Lesson 3 (day 11)**

- Start: Review the coping style of the past 5 days.
- Proceed: Assess the consequences of coping manners.
- Transform:
  Elucidate the 6 facets of PF and PI of ACT with different examples.
  Mindfulness practice: mindful breathing.
- Integrate: Choose one facet of PF and use it as a coping strategy in the following 5 days.
- Homework: Practice mindfulness (breathing) daily until the next lesson.

**Lesson 4 (day 16)**

- Start: Clarify which facet of PI is most likely to be their previous coping manners.
- Proceed: Think about the changes in their coping manners after having joined this study.
- Transform:
  Elucidate the 6 facets of PF and PI of ACT with different examples.
  Mindfulness practice: mindful walking.
- Integrate: Choose one facet of PF and use it as a coping strategy in the following 5 days.
- Homework: Practice mindfulness (walking) daily until the next lesson.

**Lesson 5 (day 21)**

- Start: Clarify which facet of PF is most likely to be their previous coping manners.
- Proceed: Think about the changes in their coping manners after having joined this study.
- Transform:
  Elucidate the 6 facets of PF and PI of ACT with different examples.
  Mindfulness practice: Metta meditation.
- Integrate: Choose one facet of PF and use it as a coping strategy in the following 5 days.
- Homework: Practice mindfulness (Metta meditation) daily until the next lesson.

**Lesson 6 (day 26)**

- Start: Determine medium and long-term objectives.
- Proceed: Think about how to achieve objectives.
- Transform:
  Elucidate the 6 facets of PF and PI of ACT with different examples.
  Mindfulness practice: breathing space: the door to actions.
- Integrate:
  Use the 6 facets of PF in their life.
  Actions are a must.
- Homework: Practice mindfulness (the door to actions) daily until the next lesson.

### Measures

#### Outcome Measures

##### Depression, Anxiety, and Stress

The DASS-21 [67] consists of 3 self-report subscales assessing depression, anxiety, and stress. Participants indicate their experience over the past week using a 4-point Likert scale (0=not at all and 3=very much so). Each subscale score is calculated by summing the scores of its 7 items and multiplying by 2, resulting in a range of 0 to 42, where higher scores reflect greater severity of the respective emotional state. Cutoff scores for mild levels of distress are depression subscale ≥10, anxiety subscale ≥8, and stress subscale ≥15 [65,66]. The DASS-21 is widely used in both clinical practice and research (T1: Cronbach α=0.71, McDonald ω=0.73; T2: Cronbach α=0.86, McDonald ω=0.88; T3: Cronbach α=0.87, McDonald ω=0.88).

##### About OCSs

The 18-item revised Obsessive-Compulsive Inventory [68] was used to assess the frequency and pain of individual OCSs (eg, “I always check the doors, windows, and drawers again and again”). It consists of 6 dimensions: washing, checking, obsessing, neutralizing, ordering, and hoarding. Participants were asked to rate the frequency of OCSs that had occurred in the past month (0 being “never” and 4 being “almost always”) and how painful they had been (0 being “not painful at all” and 4 being “extremely painful”). The total score ranged from 0 to 72, with higher scores reflecting greater symptom severity (T1: Cronbach α=0.84, McDonald ω=0.74; T2: Cronbach α=0.91, McDonald ω=0.91; T3: Cronbach α=0.91, McDonald ω=0.91).

##### About PI

The Multidimensional Psychological Flexibility Inventory [69] contains 2 subscales: PF and PI. Each subscale contains six dimensions, corresponding to 6 facets of PF (eg, “I try to reconcile my negative thoughts and feelings instead of fighting them”) and 6 facets of PI, respectively (eg, “When I have negative thoughts or emotions, it's hard to get out of them”), with a total of 60 items rated on a 1 to 6 scale. Scores for each subscale range from 30 to 180, where higher scores indicate greater PI or PF. The content validity, structure validity, and scale validity of the total scale and each subscale all meet the statistical requirements [69]. The PI subscale was adopted in this study (T1: Cronbach α=0.88, McDonald ω=0.88; T2: Cronbach α=0.92, McDonald ω=0.92; T3: Cronbach α=0.92, McDonald ω=0.92).

#### Statistical Analyses

Statistical analyses were conducted using SPSS (version 25; IBM Corp). The chi-squared test was used to analyze qualitative variables (eg, demographic data). For continuous variables, the independent-sample *t* test (2-tailed) and 1-way ANOVA were applied. Analyses of the intervention effects were executed with repeated-measures ANOVA, which included between-group factors, within-group factors, and group×time interaction. Whenever the sphericity assumption was not met, the Greenhouse-Geisser ε (*ɛ*<0.75) was used. Bonferroni correction was applied to adjust the *P* values for multiple comparisons. Specifically, after selecting the estimated marginal means in the analysis, we checked the main effect comparisons and selected Bonferroni for the CI adjustment. Effect sizes were calculated through partial Eta² (*ηp^2^*), and the level of statistical significance for all comparisons was set at *P*<.05. An intent-to-treat analysis was applied, and the principle of last-observation-carrying-forward was used to address missing data. If no significant effect of the group×time interaction was found, no further statistical tests were conducted. Conversely, if a difference was observed, simple effect analyses were performed to assess potential interaction effects between time and group.

### Ethical Considerations

This study was approved by the Institutional Review Board of the Medical Ethics Committee of Chinese PLA General Hospital (S2023-051-02), and the authors assert that all procedures contributing to this work comply with the ethical standards of the relevant national and institutional committees on human experimentation and with the Helsinki Declaration of 1975, as revised in 2008. Informed consent was obtained from all participants, who were also informed of their right to withdraw at any time without penalty. The data collected were anonymized or de-identified, with no identifiable personal information included. There was no compensation for participation, which was entirely voluntary.

## Results

### Demographics and Baseline Data

**Figure 2:**
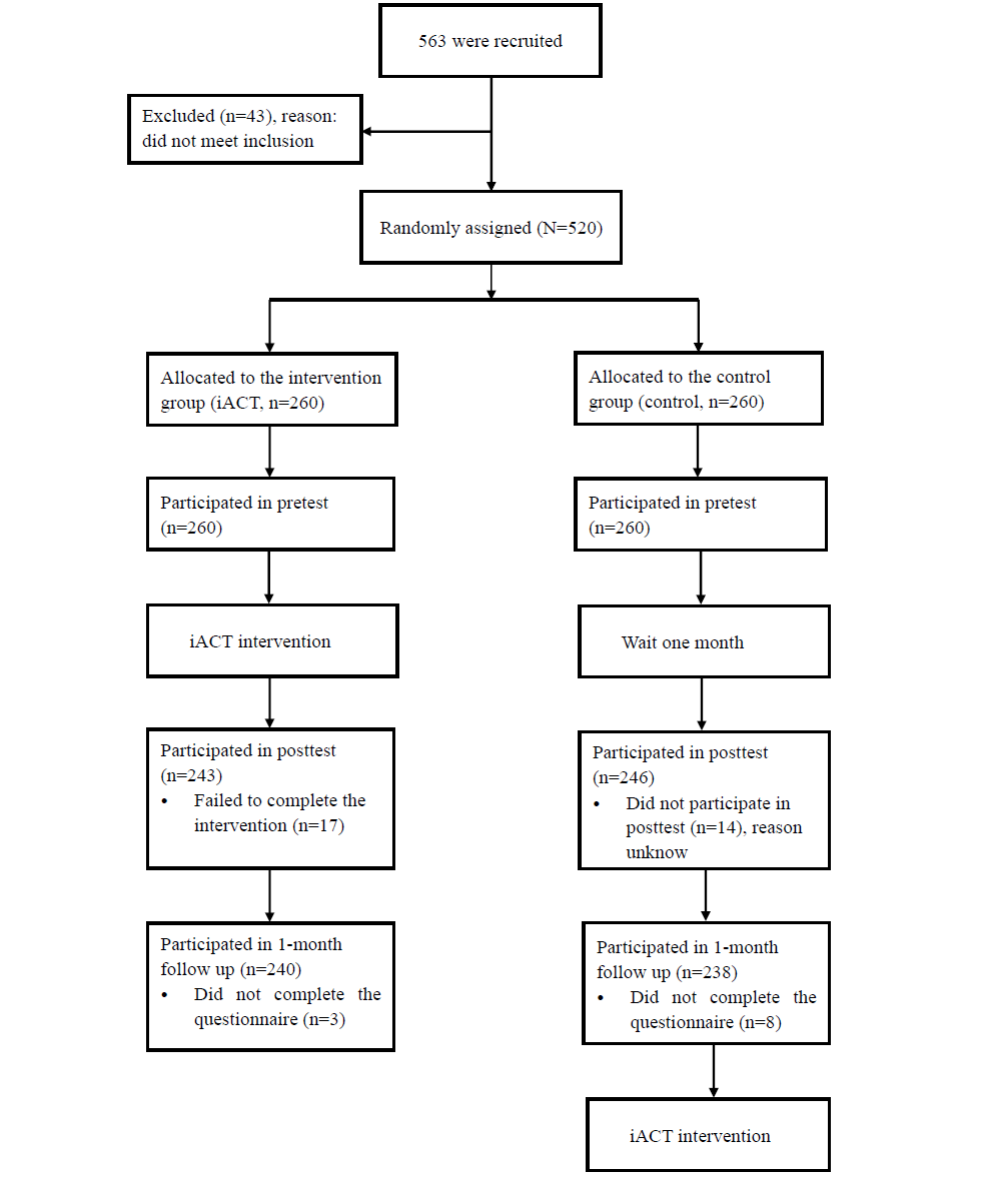
CONSORT (Consolidated Standards of Reporting Trials) participant flow diagram. iACT: internet-based acceptance and commitment therapy.

Participant characteristics between the 2 groups are presented in Table 1. There were no significant differences found between the iACT group and control group for participants’ characteristics and each outcome variable at baseline (all *P*>.05). The CONSORT (Consolidated Standards of Reporting Trials) participant flow diagram is presented in Figure 2.

However, based on the demographics analysis results, the depression, anxiety, stress, and DASS scores varied significantly among medical students with different grades (*F*_2,517_=26.28, *P*<.001; *F*_2,517_=42.36, *P*<.001; *F*_2,517_=167.79, *P*<.001; *F*_2,517_=252.91, *P*<.001). Ad hoc test results showed that final-year medical students’ scores of depression, anxiety, stress, and DASS are significantly higher than first-year students (*P*<.001), indicating they have a lower level of mental health status.

**Table 1:** Demographics and baseline data.

			iACT^a^ group (n=260)		Control group (n=260)		Chi-square or *t* test (*df*)			*P* value
**Age (years)**								0.6 (518)^b^		.54
	Mean (SD)	27.07 (3.53)		26.88 (3.09)						
	Range	23-45		22-39						
**Sex, n (%)**								0.1 (1)^c^		.71
	Male	143 (55)		147 (56.7)						
	Female	117 (45)		113 (43.3)						
**Grade, n (%)**								3.9 (2)^c^		.14
	First year	83 (34.6)		75 (31.5)						
	Second year	60 (25)		79 (33.2)						
	Final year	97 (40.4)		84 (35.3)						
DASS-21^d^ total score, mean (SD)			52.07 (10.93)		51.14 (9.76)		0.9 (518)^b^			.33
Depression score, mean (SD)			12.91 (3.16)		13.3 (2.35)		–1.5 (518)^b^			.12
Anxiety score, mean (SD)			15.38 (5.3)		14.55 (4.71)		1.8 (518)^b^			.07
Stress score, mean (SD)			23.78 (7.67)		23.29 (7.15)		0.7 (518)^b^			.48
PI^e^ score, mean (SD)			102.75 (22.81)		101.3 (2)		0.7 (518)^b^			.46
OCS^f^ score, mean (SD)			46.03 (8.31)		45.06 (8.86)		1.2 (518)^b^			.22

^a^iACT: internet-based acceptance and commitment therapy.

^b^*t* test

^c^Chi-square test

^d^DASS-21: 21-item Depression Anxiety Stress Scale.

^e^PI: psychological inflexibility.

^f^OCS: obsessive-compulsive symptom.

### Effect of the iACT Intervention on Outcome Variables

#### Effect of the iACT Intervention on DASS-21 Score

**Figure 3:**
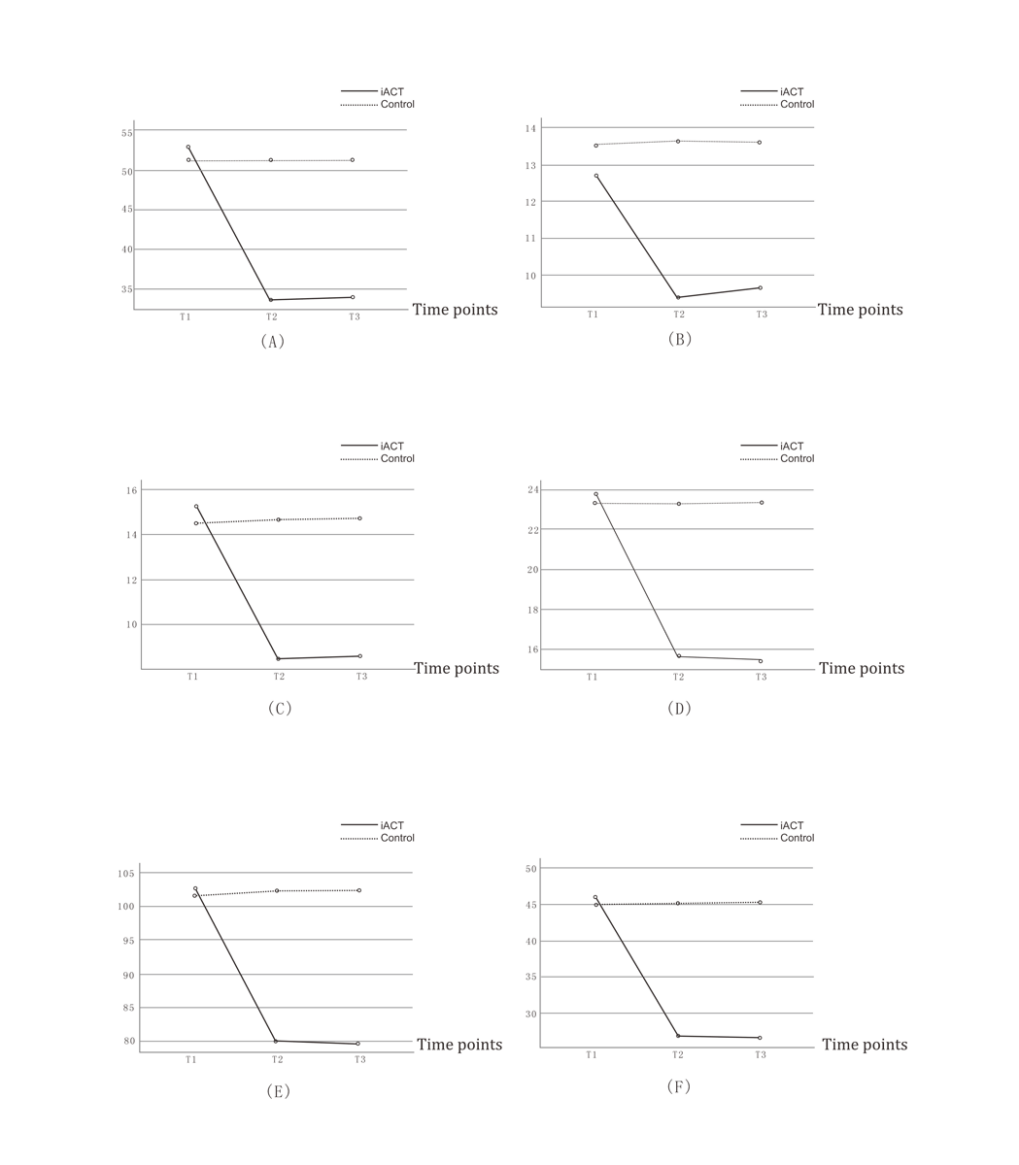
Changes of outcome variable scores over time: (A) DASS-21 total score, (B) depression score, (C) anxiety score, (D) stress score, (E) PI score, and (F) OCS score. iACT: internet-based acceptance and commitment therapy; DASS: Depression Anxiety Stress Scale; PI: psychological inflexibility; OCS: obsessive-compulsive symptom; TI: preintervention measurement; T2: postintervention measurement; T3: follow-up measurement.

As shown in Table S2 in Multimedia Appendix 1 and Figure 3A-D, the analysis revealed significant effects of time, group, and group×time interactions on the DASS-21 total score and its subscales. Participants in the intervention group demonstrated lower scores in total DASS-21, depression, anxiety, and stress compared to the control group after the intervention. Specifically, the simple effect analysis revealed that DASS-21 total score, depression score, anxiety score, and stress score were significantly lower in the intervention group at T2 and T3 (all *P*<.001), while there was no significant difference between the 3 measurements in the control group (all *P*>.05). The results of simple effect analysis between the 2 groups showed that there was no significant difference at T1 between the 2 groups, while there were significant differences at T2 and T3 (all *P*<.001). Overall, after the intervention, participants in the intervention group progressed toward a better depression, anxiety, and stress status, suggesting the intervention was effective and the improvements were maintained during the 1-month follow-up period.

#### Effect of iACT Intervention on PI

The results presented in Table S2 in Multimedia Appendix 1 and Figure 3E indicated significant effects of time, group, and group×time interactions on PI scores. Participants in the intervention group demonstrated significantly lower levels of PI at both the postintervention (T2) and 1-month follow-up time points (T3) compared to the control group (all *P*<.001). In contrast, the control group exhibited no significant changes across the 3 measurement points (all *P*>.05). The analysis revealed that there were no significant differences between the 2 groups at baseline (T1), but significant differences emerged at T2 and T3 (all *P*<.001). Overall, following the intervention, participants in the intervention group showed improved PF, suggesting that the iACT program effectively reduced PI scores, with these improvements being maintained during the follow-up period.

#### Effect of iACT Intervention on OCSs

Table S2 in Multimedia Appendix 1 and Figure 3F revealed significant effects of time, group, and group×time interactions on OCS scores. Participants in the intervention group showed a significant reduction in OCSs at both the postintervention (T2) and 1-month follow-up time points (T3) compared to the control group (all *P*<.001). In contrast, the control group did not demonstrate any significant changes across the 3 measurement points (all *P*>.05). The analysis indicated no significant differences between the 2 groups at baseline (T1), but significant differences were observed at T2 and T3 (all *P*<.001). Overall, after the intervention, participants in the intervention group progressed toward a better OCS status, suggesting the intervention was effective in reducing OCSs, and the improvements were maintained during the 1-month follow-up period.

## Discussion

### Principal Findings

This study developed and implemented a novel intervention program, iACT 2.0, rooted in ACT. We examined its suitability for online delivery and its effectiveness in improving mental health compared to a waitlist control group. Our findings indicate that the iACT 2.0 intervention significantly reduced symptoms of mental health distress, including depression, anxiety, stress, and OCSs. Moreover, it led to improvements in PF among Chinese medical students experiencing at least mild levels of depression, anxiety, and stress. These positive effects were sustained at the 1-month follow-up assessment.

A unique aspect of our study is the development of a new intervention program based on ACT, which offers several advantages. First, it integrates cultural and spiritual elements, using the Bagua diagram from traditional Chinese Daoism to illustrate dynamic thinking patterns, helping participants grasp core intervention principles within a familiar context. Second, each lesson follows the 4 steps of “启-承-转-合 (starting, proceeding, transforming, integrating)” to teach the 6 facets of PF and PI. The “启” step introduces participants to the session and sets goals. The “承” step encourages participants to review the past and connect with the present. The “转” step suggests the participants to undergo the transformation and seek change. The “合” step guides participants in integrating session content into actionable steps. This structured approach enhances understanding and acceptance of the intervention content. Third, our program provides participants with a solution from the first lesson, which attracts them to complete the 6 lessons, thereby improving adherence and retention (with a drop rate of 20/260, 7.7% in the iACT group). Fourth, each lesson is a further explanation of the 6 facets, step by step, with the whole process being a kind of recurrent reinforcement. Throughout the 6 lessons, the contents are reviewed, consolidated, and improved repeatedly, enabling participants to master ACT and gain insights. Fifth, each lesson is followed by homework, such as practicing targeted daily mindfulness and selected facet practice in the following 5 days before the next lesson, which could promote the intervention’s effectiveness. Lastly, our program gives participants more autonomy to begin with the most inflexible facet and then guides them to explore their internal resources to proceed with the whole process. Overall, our intervention aims to maximize the benefits for participants in a limited time as much as possible. It could be applied to one-time psychological assistance and consulting, offering a good opportunity to address common mental health conditions in medical students while simultaneously reducing the burden on traditional counseling and services.

In recent years, there has been increasing research focused on interventions to improve the mental health of medical students. However, existing studies are limited by small sample sizes and lack of representativeness. For instance, one study included only 91 medical students from An-Najah National University, Palestine, in a cognitive behavioral therapy program for mental health status [70]. Another study involved 120 undergraduate nursing students in China to examine the effectiveness of an online mindfulness intervention course on their mental health [71]. By contrast, our study included 478 postgraduate medical students in China, and the results demonstrated a significant reduction in mean values of depression, anxiety, stress, total DASS-21, OCS score, and PI score compared to a waitlist control group after the intervention. Importantly, no personal therapist contact was incorporated during our intervention, allowing us to isolate the influence of therapist instruction and demonstrate the effectiveness of a self-help model. Although previous research has not reached a consensus regarding the role of therapist support during online interventions, with some studies finding unsupported interventions did not lead to significant changes in PF, and others reporting that web-based, stand-alone treatments are comparably effective to therapist-assisted interventions [54,72], our findings contribute to the growing research on brief iACT interventions. Our findings also have cost implications, given that personnel costs often constitute the largest proportion of online interventions and face-to-face consulting costs [73]. If the developed self-help intervention is effective without the assistance of paid personnel, the participant cost in research and psychotherapy costs in real-world practice may be reduced.

Our study revealed that final-year postgraduate medical students reported significantly lower levels of positive mental health and higher levels of mental health distress compared to first-year students, which is consistent with the findings of a recent study by Wimberly et al [74]. These results provide further evidence for the association between grade level and mental health distress among medical students. They also suggest that university administrators and educators should pay more attention to the mental health of final-year students and consider providing additional psychological support resources to this particular cohort.

### Limitations and Future Research

Overall, this study could make a significant contribution to promoting online psychotherapy for improving medical students’ mental well-being, with strengths including the self-help iACT intervention formulation, follow-up study, and relatively large sample size. However, it also had several limitations. First, the participants in our study were postgraduate medical students, who are highly educated and familiar with modern technology, thereby limiting the representativeness of the sample. Future research should investigate the program’s effectiveness among populations lacking digital literacy or within clinical demographics. Second, the use of self-report scales may potentially cause response bias, participants may also lose the patience to fill in carefully. To address this, future research could use adaptive algorithms to customize differentiated scales for different participants, allowing for real-time jumping to the most relevant items through the interaction with participants’ answers. Third, this study integrated the entire 6 facets of ACT into the core component of the intervention and examined its efficacy, future research should explore and examine which specific facet is most important during the intervention. Finally, this study did not explore intervention-related placebo effects, which should be considered in future research.

### Conclusion

In conclusion, this study demonstrates that the newly developed iACT 2.0 effectively alleviates depression, anxiety, stress, OCSs, and PI among medical students experiencing at least mild levels of psychological distress. Furthermore, the observed positive effects were sustained at the 1-month follow-up, suggesting the program’s viability as a practical intervention for addressing prevalent mental health issues. This offers a promising alternative to conventional counseling services, potentially alleviating their burden.

## Appendix

Multimedia Appendix 1 Supplementary materials.

Multimedia Appendix 2 CONSORT-eHEALTH checklist (V 1.6.1).

## Abbreviations

ACT: acceptance and commitment therapy
CONSORT: Consolidated Standards of Reporting Trials
DASS-21: 21-item Depression Anxiety Stress Scale
iACT: internet-based acceptance and commitment therapy
iACT 2.0: internet-based, self-help, acceptance and commitment therapy program
OCD: obsessive-compulsive disorder
OCS: obsessive-compulsive symptom
PF: psychological flexibility
PI: psychological inflexibility

## Data Availability

The datasets generated or analyzed during this study are available from the corresponding author on reasonable request.
