# Supplementary material for: Effectiveness of an Internet-Based Self-Help Acceptance and Commitment Therapy Program on Medical Students’ Mental Well-Being: Follow-Up Randomized Controlled Trial
Source: J Med Internet Res. 2024 Dec 4;26:e50664. doi: 10.2196/50664 (PMC11656501; doi:10.2196/50664)
Supplement: Multimedia Appendix 1 [file jmir_v26i1e50664_app1.docx]

**Table S1.** Detailed protocol of iACT 2.0 program.

| Time | Content Summary |
| --- | --- |
| lesson 1 (day1) | **1.Start** Ask participants to determine today's objectives. **2. Proceed** Ask participants to review their previous coping styles. **3. Transform** A. Elucidate of the 6 facets of PF and PI of ACT. (a) Explain the concept of Experiential Avoidance, Acceptance, Cognitive Fusion, Defusion, Dominance of the Conceptualized Past and Feared Future, Contact with the Present Moment, Attachment to the Conceptualized Self, Self as Context, Lack of Values, Values, Inaction, and Committed Action. (b) Introduce ways of being acceptance, achieving cognitive defusion, contacting with the present moment, achieving self as context, clarifying values, and acting. B. Mindfulness Practice: Body Scan in Mindfulness. **4.Integrate** Encourage participants to choose one facet of PF that works best for him/her and use it as coping strategy in the following 5 days .  **Homework** Ask participants to practice Mindfulness (Body Scan) with a daily basis until next lesson. |
| lesson 2 (day6) | **1. Start** Ask participants to review the coping style of the past 5 days. **2. Proceed** Ask participants to think about the long-term effects of previous coping manners that they used before this study. **3. Transform** A. Use different examples or stories from the last lesson to elucidate the 6 facets of PF and PI of ACT. (a) Explain the concept of Experiential Avoidance, Acceptance, Cognitive Fusion, Defusion, Dominance of the Conceptualized Past and Feared Future, Contact with the Present Moment, Attachment to the Conceptualized Self, Self as Context, Lack of Values, Values, Inaction, and Committed Action. (b) Introduce ways of being acceptance, achieving cognitive defusion, contacting with the present moment, achieving self as context, clarifying values, and acting. B. Mindfulness Practice: Body Stretch. **4.Integrate** Encourage participants to choose one facet of PF that works best for him/her and use it as coping strategy in the following 5 days.  **Homework** Ask participants to practice Mindfulness (Body Stretch) with a daily basis until next lesson. |
| lesson 3 (day11) | **1. Start** Ask participants to review the coping style of the past 5 days. **2. Proceed** Ask participants to assess the consequences of coping manners that they used before this study. **3. Transform** A. Use different examples or stories from the previous 2 lessons to elucidate the 6 facets of PF and PI of ACT. (a) Explain the concept of Experiential Avoidance, Acceptance, Cognitive Fusion, Defusion, Dominance of the Conceptualized Past and Feared Future, Contact with the Present Moment, Attachment to the Conceptualized Self, Self as Context, Lack of Values, Values, Inaction, and Committed Action. (b) Introduce ways of being acceptance, achieving cognitive defusion, contacting with the present moment, achieving self as context, clarifying values, and acting. B. Mindfulness Practice: Mindful Breathing. **4.Integrate** Encourage participants to choose one facet of PF that works best for him/her and use it as coping strategy in the following 5 days.  **Homework** Ask participants to practice Mindfulness (Breathing) with a daily basis until next lesson. |
| lesson 4 (day16) | **1. Start** Ask participants to clarify which facet of PI is most likely to their coping manners that they used before this study. **2. Proceed** Ask participants to think about the changes of their coping manners after joining this study. **3. Transform** A. Use different examples or stories from the previous 3 lessons to elucidate the 6 facets of PF and PI of ACT. (a) Explain the concept of Experiential Avoidance, Acceptance, Cognitive Fusion, Defusion, Dominance of the Conceptualized Past and Feared Future, Contact with the Present Moment, Attachment to the Conceptualized Self, Self as Context, Lack of Values, Values, Inaction, and Committed Action. (b) Introduce ways of being acceptance, achieving cognitive defusion, contacting with the present moment, achieving self as context, clarifying values, and acting. B. Mindfulness Practice: Mindful Walking. **4.Integrate** Encourage participants to choose one facet of PF that works best for him/her and use it as coping strategy in the following 5 days.  **Homework** Ask participants to practice Mindfulness (Walking) with a daily basis until next lesson. |
| lesson 5 (day21) | **1. Start** Ask participants to clarify which facet of PF is most likely to their coping manners that they used before this study. **2. Proceed** Ask participants to think about the changes of their coping manners after joining this study. **3. Transform** A. Use different examples or stories from the previous 4 lessons to elucidate the 6 facets of PF and PI of ACT. (a) Explain the concept of Experiential Avoidance, Acceptance, Cognitive Fusion, Defusion, Dominance of the Conceptualized Past and Feared Future, Contact with the Present Moment, Attachment to the Conceptualized Self, Self as Context, Lack of Values, Values, Inaction, and Committed Action. (b) Introduce ways of being acceptance, achieving cognitive defusion, contacting with the present moment, achieving self as context, clarifying values, and acting. B. Mindfulness Practice: Metta Meditation. **4.Integrate** Encourage participants to choose one facet of PF that works best for him/her and use it as coping strategy in the following 5 days.  **Homework** Ask participants to practice Mindfulness (Metta Meditation) with a daily basis until next lesson. |
| lesson 6 (day26) | **1. Start** Ask participants to determine medium and long-term objectives. **2. Proceed** Ask participants to think how to achieve their objectives. **3. Transform** A. Use different examples or stories from the previous 5 lessons to elucidate the 6 facets of PF and PI of ACT. (a) Explain the concept of Experiential Avoidance, Acceptance, Cognitive Fusion, Defusion, Dominance of the Conceptualized Past and Feared Future, Contact with the Present Moment, Attachment to the Conceptualized Self, Self as Context, Lack of Values, Values, Inaction, and Committed Action. (b) Introduce ways of being acceptance, achieving cognitive defusion, contacting with the present moment, achieving self as context, clarifying values, and acting. B. Mindfulness Practice: Breathing Space: the Door to Actions. **4.Integrate** A. Encourage participants use the 6 facets of Psychological Flexibility in their life.  B. Tell participants conscious Actions is a must after the intervention. **Homework** Ask participants to practice Mindfulness (the Door to Actions) with a daily basis until next lesson. |

**Table S2.** Repeated measures ANOVA results.^a^

|  | pre-measurement(T1) | post-measurement(T2) | 1-month follow-up(T3) |  | | |  |
| --- | --- | --- | --- | --- | --- | --- | --- |
|  |  |  |  |  |  |  |  |
|  | *M*±*SD* | *M*±*SD* | *M*±*SD* | *F* value^b^ *(df)* | *P* value | Partial *ηp² (90% CI)* |  |
| **DASS Total** |  |  |  |  |  |  |  |
| intervention group | 52.07±10.93 | 33.54±15.80 | 33.78±15.94 |  |  |  |  |
| control group | 51.14±9.76 | 51.19±14.36 | 51.27±11.77 |  |  |  |  |
| main effect(time) |  |  |  | 73.96 (2, 1038) | <.001 | 0.13 (0.09, 0.18) |  |
| main effect(group) |  |  |  | 273.79 (1, 518) | <.001 | 0.37 (0.31, 0.41) |  |
| interaction effect(group×time) |  |  |  | 75.38 (2, 1038) | <.001 | 0.14 (0.09, 0.18) |  |
| **Depression** |  |  |  |  |  |  |  |
| intervention group | 12.91±3.16 | 9.40±5.33 | 9.66±5.66 |  |  |  |  |
| control group | 13.30±2.35 | 13.35±4.08 | 13.39±4.52 |  |  |  |  |
| main effect(time) |  |  |  | 22.90 (2, 1038) | <.001 | 0.05 (0.02, 0.08) |  |
| main effect(group) |  |  |  | 142.78 (1, 518) | <.001 | 0.23 (0.18, 0.28) |  |
| interaction effect(group×time) |  |  |  | 24.73 (2, 1038) | <.001 | 0.05 (0.02, 0.08) |  |
| **Anxiety** |  |  |  |  |  |  |  |
| intervention group | 15.38±5.30 | 8.48±5.19 | 8.65±4.92 |  |  |  |  |
| control group | 14.55±4.71 | 14.60±5.55 | 14.57±5.24 |  |  |  |  |
| main effect(time) |  |  |  | 68.13 (2, 1038) | <.001 | 0.13 (0.08, 0.17) |  |
| main effect(group) |  |  |  | 191.54 (1, 518) | <.001 | 0.29 (0.23, 0.34) |  |
| interaction effect(group×time) |  |  |  | 69.68 (2, 1038) | <.001 | 0.13 (0.08, 0.17) |  |
| **Stress** |  |  |  |  |  |  |  |
| intervention group | 23.78±7.67 | 15.66±13.67 | 15.47±13.31 |  |  |  |  |
| control group | 23.29±7.15 | 23.24±11.72 | 23.31±8.41 |  |  |  |  |
| main effect(time) |  |  |  | 23.38 (2, 1038) | <.001 | 0.05 (0.02, 0.08) |  |
| main effect(group) |  |  |  | 80.59 (1, 518) | <.001 | 0.15 (0.10, 0.19) |  |
| interaction effect(group×time) |  |  |  | 23.20 (2, 1038) | <.001 | 0.05 (0.02, 0.08) |  |
| **Psychological Inflexibility** |  |  |  |  |  |  |  |
| intervention group | 102.75±22.81 | 79.95±19.04 | 79.71±18.79 |  |  |  |  |
| control group | 101.30±20 | 102.43±22.54 | 102.53±22.44 |  |  |  |  |
| main effect(time) |  |  |  | 41.68 (2, 1038) | <.001 | 0.08 (0.05, 0.12) |  |
| main effect(group) |  |  |  | 182.52 (1, 518) | <.001 | 0.28 (0.22, 0.33) |  |
| interaction effect(group×time) |  |  |  | 51.17 (2, 1038) | <.001 | 0.10 (0.06, 0.14) |  |
| **Obsessive Compulsive Symptom** |  |  |  |  |  |  |  |
| intervention group | 46.03±8.31 | 26.78±9.12 | 26.61±8.38 |  |  |  |  |
| control group | 45±8.86 | 45.18±8.36 | 45.24±8.32 |  |  |  |  |
| main effect(time) |  |  |  | 205.24 (2, 1038) | <.001 | 0.30 (0.25, 0.35) |  |
| main effect(group) |  |  |  | 672.04 (1, 518) | <.001 | 0.59 (0.54, 0.62) |  |
| interaction effect(group×time) |  |  |  | 211.69 (2, 1038) | <.001 | 0.31 (0.25, 0.36) |  |

**^a^** Effect sizes were calculated through partial Eta² (*ηp²*). *ηp²* ≤ 0.05 corresponds to a small effect, *ηp²* [0.05; 0.25] to a medium effect, *ηp²* [0.25; 0.50] to a large effect, and *ηp²* > 0.50 to a very large effect. F-value: Ratio of variance between groups to variance within groups, used in ANOVA to assess statistical significance of differences.


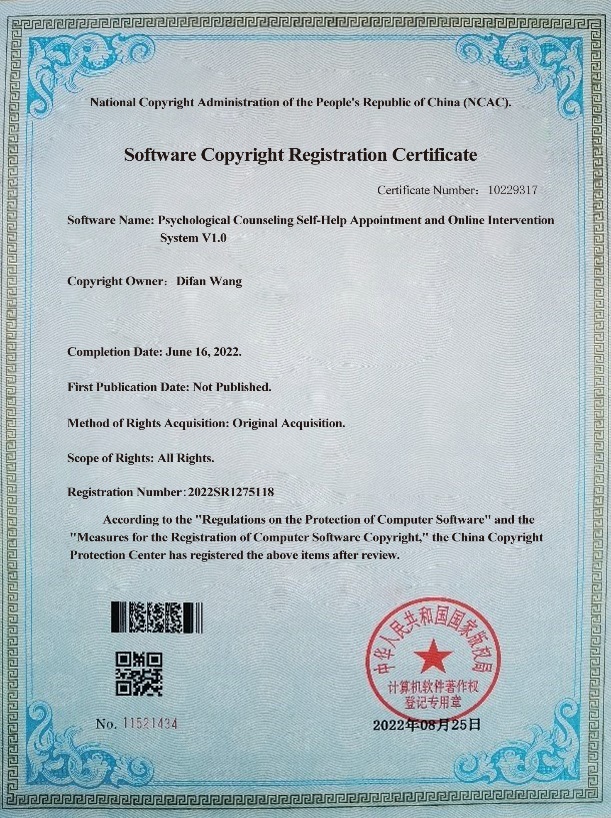


(a)


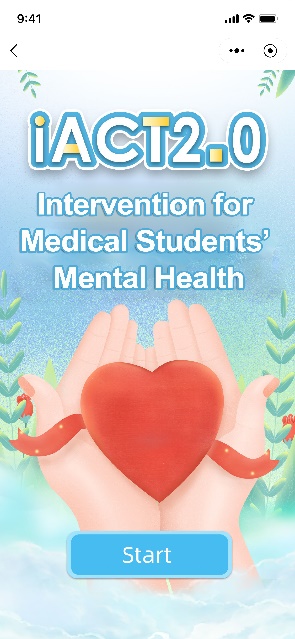

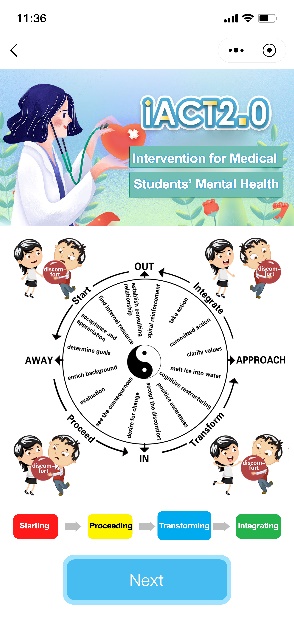

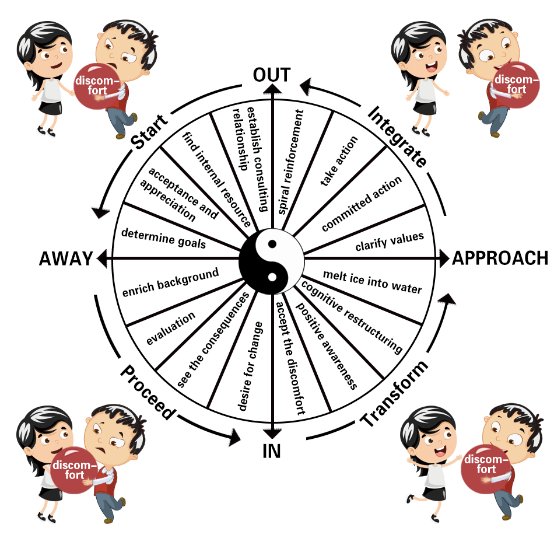


(b) (c)

**Figure S1.** English version of Starting-Proceeding-Transforming-Integrating.
